# Identification of clinically relevant T cell receptors for personalized T cell therapy using combinatorial algorithms

**DOI:** 10.1038/s41587-024-02232-0

**Published:** 2024-05-07

**Authors:** Rémy Pétremand, Johanna Chiffelle, Sara Bobisse, Marta A. S. Perez, Julien Schmidt, Marion Arnaud, David Barras, Maria Lozano-Rabella, Raphael Genolet, Christophe Sauvage, Damien Saugy, Alexandra Michel, Anne-Laure Huguenin-Bergenat, Charlotte Capt, Jonathan S. Moore, Claudio De Vito, S. Intidhar Labidi-Galy, Lana E. Kandalaft, Denarda Dangaj Laniti, Michal Bassani-Sternberg, Giacomo Oliveira, Catherine J. Wu, George Coukos, Vincent Zoete, Alexandre Harari

**Affiliations:** 1 Ludwig Institute for Cancer Research, Lausanne Branch, Department of Oncology, Lausanne University Hospital (CHUV) and University of Lausanne (UNIL), Agora Cancer Research Center, Lausanne, Switzerland; 2 Center for Cell Therapy, CHUV-Ludwig Institute, Lausanne, Switzerland; 3 Molecular Modelling Group, Swiss Institute of Bioinformatics (SIB), Lausanne, Switzerland; 4 Center of Experimental Therapeutics, Lausanne University Hospital (CHUV), Lausanne, Switzerland; 5 Department of Medicine and Center of Translational Research in Onco-Hematology, Faculty of Medicine, University of Geneva, Swiss Cancer Center Leman, Geneva, Switzerland; 6 Division of Clinical Pathology, Department of Diagnostics, Hôpitaux Universitaires de Genève, Geneva, Switzerland; 7 Department of Oncology, Hôpitaux Universitaires de Genève, Geneva, Switzerland; 8 Dana-Farber Cancer Institute, Boston, MA USA; 9 Harvard Medical School, Boston, MA USA; 10 Broad Institute of MIT and Harvard, Cambridge, MA USA; 11 Immuno-oncology Service, Department of Oncology, Lausanne University Hospital, Lausanne, Switzerland

**Keywords:** Tumour immunology, Applied immunology, Immunotherapy, Translational research, Computational models

## Abstract

A central challenge in developing personalized cancer cell immunotherapy is the identification of tumor-reactive T cell receptors (TCRs). By exploiting the distinct transcriptomic profile of tumor-reactive T cells relative to bystander cells, we build and benchmark TRTpred, an antigen-agnostic in silico predictor of tumor-reactive TCRs. We integrate TRTpred with an avidity predictor to derive a combinatorial algorithm of clinically relevant TCRs for personalized T cell therapy and benchmark it in patient-derived xenografts.

## Abstract (web summary)

Tumor-reactive T cell receptors are predicted based on single-cell RNA sequencing and single-cell T cell receptor sequencing.

## Main

Adoptive cell transfer (ACT) of tumor-infiltrating lymphocytes (TILs) is a personalized immunotherapy approach with demonstrated superiority over second-line checkpoint blockade immunotherapy in patients with melanoma^1,2^. Recent studies indicate that the frequency of tumor-reactive T cells (TRTs) in melanoma tumors used to expand TILs, and their number in cognate TIL products infused to patients are important determinants of TIL ACT efficacy^3,4^. Moreover, it has been hypothesized that the paucity of TRTs in other solid tumors can explain the lower success rate of TIL therapy in these patients^5^. Alternatively, the use of TCR-engineered T cells has shown promise for treating solid tumors^6^. However, when personalized, this approach faces the challenge of including TCRs with untested tumor specificity or insufficient affinity, stressing the need to identify clinically relevant TCRs^6,7^.

Recent progress in single-cell RNA and TCR sequencing (scRNA-seq and scTCR-seq) technologies enabled the exploration of the unique transcriptomic profile of intratumoral neoantigen-specific and tumor-reactive T cells^8–15^, providing an opportunity to derive predictors of private TRTs capable of reliably identifying tumor-reactive over bystander TILs, thus opening the door for the development of personalized TCR-based therapies. However, as of today, any practical application has been hampered by the lack of a rigorous evaluation framework and robust benchmarking against external datasets^8,12,14^. Moreover, predicting all TRTs indiscriminately may result in the selection of clinically suboptimal low-avidity TCRs (occurring also for some neoantigen-specific clonotypes)^14,16^.

**Fig. 1:**
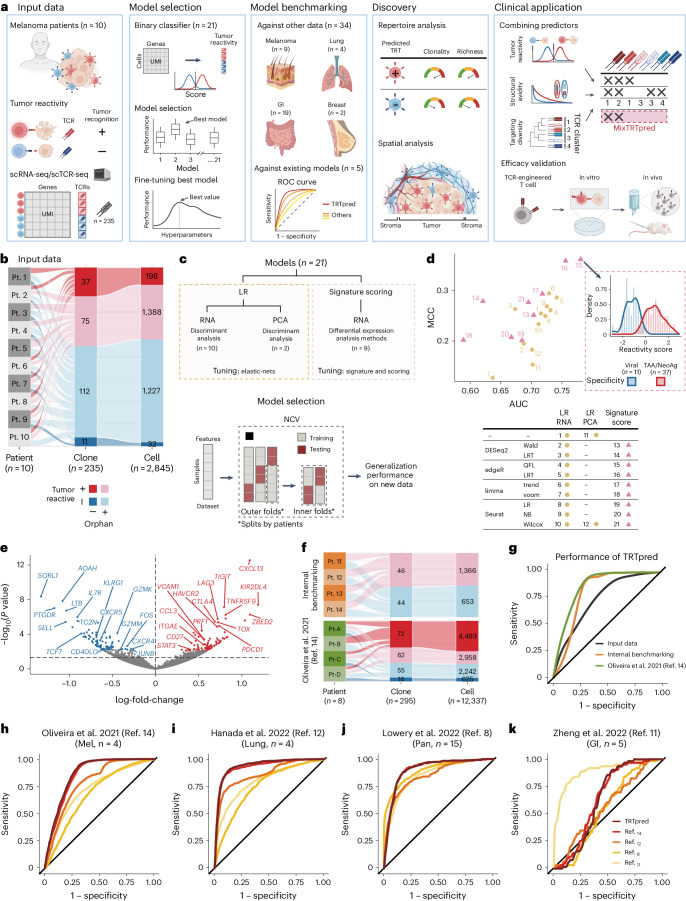
TRTpred, a sensitive in silico predictor of tumor-reactive clonotypes. **a**, Illustration of TRTpred design, benchmarking and applications. The final algorithm, MixTRTpred, combines TRTpred with a structural avidity predictor^16^ and TCRpcDist^18^, a TCR clustering algorithm. **b**, Alluvial plot showing the fractions of cells and clones annotated as tumor-reactive or non-tumor-reactive (orphan or antigen (Ag)-specific) within the input data (*n* = 10 patients with melanoma). **c**, Top, design of the 12 LR and 9 signature scoring models with their hyperparameters (Methods). Bottom, model selection framework estimating the generalization performance of the model through an LOPO NCV. **d**, Evaluation of the 12 LR (yellow circles) and 9 signature scoring (pink triangles) binary classifiers in the function of MCC and the AUC (Supplementary Table 2). The panel shows the distribution of the best model scores for tumor antigen-specific (red) and viral-specific (blue) clones. **e**, Volcano plot displaying the differential gene expression analysis comparing tumor-reactive and non-tumor-reactive cells. The 90 upregulated (red) and downregulated (blue) genes obtained by edgeR-QFL are shown (Supplementary Table 4). The *P* values are calculated using the two-sided quasi-likelihood *F*-test in the edgeR package and are corrected for multiple testing using the Benjamini–Hochberg procedure. **f**, Alluvial plots showing the fractions of cells and clones annotated as tumor-reactive or non-tumor-reactive (orphan or Ag-specific) within internal (top) and external (bottom, ref. ^14^) benchmarking data. **g**, ROC curve of TRTpred applied on the input data (black), and the internal (orange) and external (green, ref. ^14^) benchmarking data. **h**–**k**, ROC curves of TRTpred and four CD8^+^ TIL tumor-reactive predictive signatures (refs. ^8,11,12,14^) applied to the four datasets: ref. ^14^ (melanoma, *n* = 4) (**h**), ref. ^12^ (lung, *n* = 4) (**i**), ref. ^8^ (*n* = 1 melanoma, *n* = 2 breast and *n* = 12 GI) (**j**) and ref. ^11^ (GI, *n* = 5) (**k**). All AUCs are reported in Extended Data Fig. 3e. Pt, patient; TAA, tumor-associated antigen; UMI, unique molecular identifier; PCA, principal component analysis; Mel, melanoma; Pan, pan-cancer. Source data

In this study, we introduce TRTpred, an antigen-agnostic in silico TRT predictor developed and extensively benchmarked within a machine learning framework. We demonstrate its superiority compared with existing predictive TRT signatures in datasets from different tumor indications^8,11,12,14,17^. We also applied TRTpred to successfully explore the immune repertoire of tumor-reactive TILs across various tumor indications and microenvironments. Finally, by integrating a high-avidity TCR predictor^16^ and a TCR clustering algorithm (TCRpcDist)^18^, we have engineered a combinatorial algorithm (referred to as MixTRTpred) for the selection of clinically relevant TCRs from the pool of TRTs, which was subsequently validated in vitro and in vivo (Fig. 1a).

**Extended Data Fig. 1:**
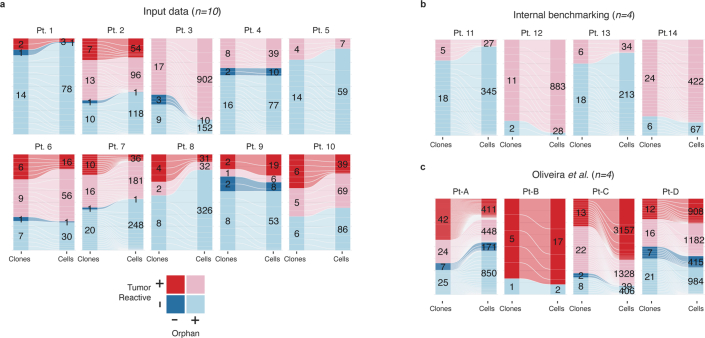
Description of internal and external benchmarking cohorts. **a**) Alluvial plot showing the fractions of cells and clones annotated as tumor-reactive or non-tumor-reactive (orphan or antigen (Ag)-specific) within the input data (n = 10 melanoma patients). **b-c**) Alluvial plots showing the fractions of cells and clones annotated as tumor-reactive or non-tumor-reactive (orphan or antigen (Ag)-specific) within the internal (**b**, *n* = 4) and external (**c**, *n* = 4, Oliveira et al.^14^) data. Source data

**Extended Data Fig. 2:**
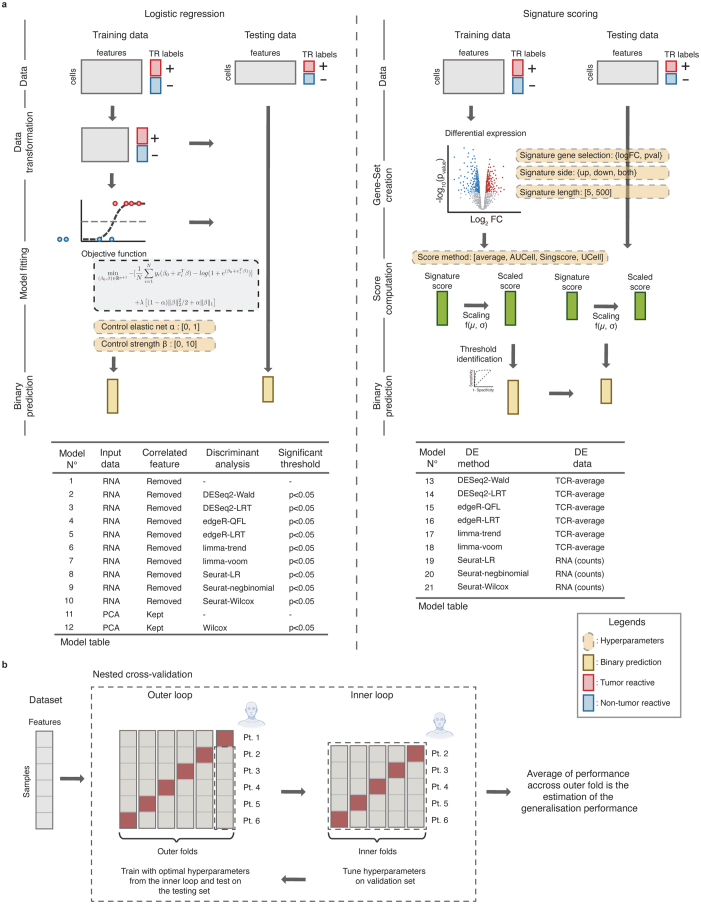
Model design and training architecture. **a**) Illustration of Logistic Regression (LR) and signature scoring models, hyperparameters and outputs (see Methods). The tables describe the 21 models. **b**) Illustration of the nested-cross-validation framework to train and evaluate the models. Here we adapt a leave-one-patient-out approach to better simulate the model performance on new data from a new patient (see Methods). In box plots, the boxes represent the median and the interquartile range (IQR), while the whiskers extend to 1.5 times the IQR.

**Extended Data Fig. 3:**
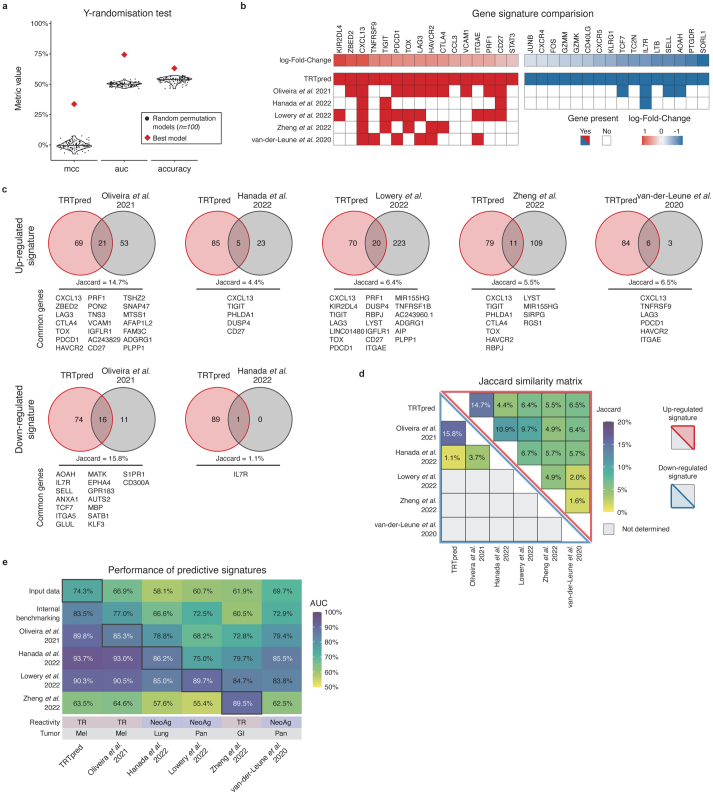
TRTpred’s validation and results. **a**) Y-random permutation tests (black points, *n* = 100) results in terms of Matthew′s Correlation Coefficient (MCC), AUC and accuracy. The red square corresponds to the best model results demonstrating its validity. **b**) Heatmap comparing relevant up and down regulated genes from TRTpred’s signature with 5 published CD8^+^ TILs tumor-reactive signatures (Oliveira et al.^14^, Hanada et al.^12^, Lowery et al.^8^, Zheng et al.^11^ and van-der-Leune et al.^17^). **c**) Venn diagrams comparing the up (top) and down (bottom) regulated genes from TRTpred signature (red) with the 5 CD8^+^ TILs tumor-reactive signatures from panel B (grey). Underneath the Venn diagrams are the Jaccard indexes and the list of common genes. **d**) Heatmap of the Jaccard similarity index matrix comparing the 6 CD8^+^ TILs tumor-reactive signatures from panels B and C. The upper and lower triangle of this matrix correspond to the up and down regulated genes, respectively. Grey boxes correspond to missing gene-sets. **e**) Heatmap of AUC performances of 6 CD8^+^ TILs tumor-reactive predictive signatures (X-axis) applied on 6 datasets (Y-axis) comprising the input data (melanoma, *n* = 10), the internal benchmarking data (melanoma, *n* = 4) and data from 4 cohorts: Oliveira et al.^14^ (melanoma, *n* = 4), Hanada et al.^12^ (lung, *n* = 4), Lowery et al.^8^ (*n* = 1 melanoma, *n* = 2 breast and *n* = 12 GI) and Zheng et al.^11^ (GI, *n* = 5). The performance of each model on its original data is highlighted. Source data

To build TRTpred, we took advantage of 235 CD8^+^ clonotypes, annotated as tumor-reactive (*n* = 112) or non-tumor-reactive (*n* = 123), curated from ten patients with metastatic melanoma^3,19^ (Fig. 1b, Extended Data Fig. 1a and Supplementary Table 1). Bona fide tumor-reactive TCRs were defined as those reacting against autologous tumors regardless of their specific antigenic targets, as previously reported^20,21^, although some specificities were also experimentally validated for some clonotypes (Supplementary Table 1). Using both their scTCR-seq and scRNA-seq profiles, we trained a suite of 21 binary classifiers including logistic regression (LR) and signature scoring methods, which underwent fine-tuning (Methods, Fig. 1c and Extended Data Fig. 2a). The models were evaluated using the leave-one-patient-out (LOPO) nested cross-validation (NCV) framework, providing insights into their generalization performance when faced with unseen data from new patients (Methods and Extended Data Fig. 2b). While LR models exhibited commendable area under the curve (AUC) scores, one of the signature scoring models (edgeR-QFL) emerged as the most generalizable, leading to TRTpred after training on the whole dataset (Fig. 1d and Supplementary Table 2). Y-randomization tests further validated TRTpred’s credibility against spurious learning (Extended Data Fig. 3a and Methods). As an illustration of TRTpred, we focused on clonotypes from the dataset with known specificity and observed a clear discrimination between virus-specific bystander TILs and TILs specific for tumor-associated antigens or tumor-restricted neoantigens (Fig. 1d and Supplementary Table 3). In line with previous studies, TRTpred’s signature is composed of several genes associated with exhaustion (for example, *CXCL13*, *LAG3*, *TOX*, *PDCD1* or *TNFRSF9*; Fig. 1e and Supplementary Table 4). However, the overlap between published signatures^8,11,12,14,17^ is limited and *CXCL13* is the unique consensual gene (Extended Data Fig. 3b–d).

To benchmark TRTpred, we first evaluated its performance using two independent datasets, sourced internally or externally by Oliveira et al.^14^, containing 90 and 205 CD8^+^ TRT annotated clonotypes, respectively (Fig. 1f, Extended Data Fig. 1b,c and Supplementary Table 1). TRTpred exhibited consistent performance across both sets of benchmarking data (Fig. 1g). We further benchmarked TRTpred in four distinct CD8^+^ TILs datasets from different tumor indications (ref. ^14^, melanoma; ref. ^12^, lung; ref. ^8^, pan-cancer; ref. ^11^, gastrointestinal (GI); Supplementary Table 1). TRTpred outperformed the different models^8,11,12,14^ even on their own datasets (Fig. 1h–k and Extended Data Fig. 3e), with the exception of ref. ^11^ data on which all signatures underperformed.

**Fig. 2:**
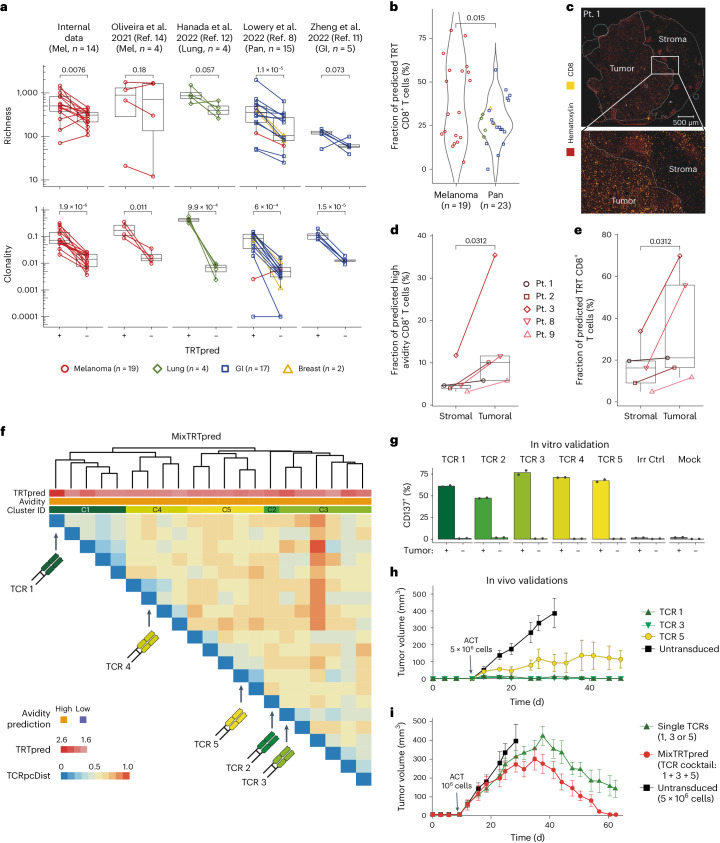
TRTpred applications for discovery of immune repertoires and validation of MixTRTpred. **a**, Richness (top) and clonality (bottom) of inferred tumor-reactive (+/−) clones using TRTpred in *n* = 5 cohorts: internal data (melanoma *n* = 14); ref. ^14^ (melanoma, *n* = 4); ref. ^11^ (GI, *n* = 5); ref. ^8^ (*n* = 1 melanoma, *n* = 2 breast and *n* = 12 GI); ref. ^12^ (lung, *n* = 4). Patients are color-coded according to the cancer type. Metrics are displayed in logarithmic scale and statistics were performed using a one-tailed *t*-test. **b**, Proportion of inferred TRT CD8^+^ T cells in melanoma (*n* = 19) and other solid tumors (*n* = 23, as described in **a**). Statistics were performed using a one-tailed Wilcoxon test. **c**, Sequential multiplexed immunohistochemistry of patient 1 with hematoxylin (red) and CD8 (yellow) staining. Upper panel, whole-tissue section; lower panel (white rectangle), magnified image. Scale bars, 500 μm and 50 μm, for the whole-tissue section and magnified images, respectively. This is a representative experiment among *n* = 5 independent patients with melanoma. **d**,**e**, Cumulative frequency of inferred high-avidity (**d**) or tumor-reactive (**e**) CD8^+^ clones identified in microdissected areas of stroma and tumor in *n* = 5 independent patients with melanoma (patients 1–5). Statistics were performed using a pairwise one-tailed Wilcoxon test. **f**, TRTpred results depicting the distance matrix of the top 20 ranked tumor-reactive among high structural avidity clones. The five clones selected are the ones with the highest tumor-reactive score in each cluster (TCRs 1–5; Supplementary Table 6), defined by hierarchical clustering. **g**, In vitro validation of the tumor reactivity of the five TCRs (TCRs 1–5) predicted through MixTRTpred through CD137 upregulation assay (mean of *n* = 2 biologically independent replicates). The color code corresponds to that of panel **f**. **h**,**i**, IL-2 NOG mice were subcutaneously engrafted with tumor cells from patient 14 followed by adoptive transfer of TCR-transduced primary CD8^+^ T cells. **h**, Tumor-bearing mice received 5 × 10^6^ CD8^+^ T cells transduced (day 11) with TCR1, TCR3 or TCR5 (Supplementary Table 6). **i**, Mice were adoptively transferred with infusion products containing 1 × 10^6^ total CD8^+^ T cells transduced (day 11) with TCR1 or TCR3 or TCR5 (single TCRs) or with a pool of 1 × 10^6^ total CD8^+^ T cells transduced with the three TCRs (TCR cocktail, 0.33 × 10^6^ CD8^+^ T cells transduced with each TCR). In **h** and **i**, 5 × 10^6^ CD8^+^ untransduced T cells were transferred as control. Data show mean ± s.e.m. of *n* = 3–5 biologically independent replicates. In box plots, the boxes represent the median and the interquartile range (IQR), while the whiskers extend to 1.5 times the IQR. ID, identification; Irr Ctrl, irrelevant control; Mel, melanoma; Pan, pan-cancer; GI, gastrointesinal. Source data

We then applied TRTpred to interrogate the tumor repertoires from 42 patients with melanoma (*n* = 19) as well as GI (*n* = 17), lung (*n* = 4) or breast (*n* = 2) cancer (Supplementary Table 1). Consistently across the different tumor types, inferred TRT repertoires were richer and more clonal than cognate bystander counterparts (Fig. 2a and Supplementary Table 5). Also, in line with our expectations based on the clinical efficacy of TIL therapy reported in melanoma versus other solid tumors^5^, a higher proportion of CD8^+^ TRTs was identified in melanoma relative to other solid tumors (Fig. 2b and Supplementary Table 5).

**Extended Data Fig. 4:**
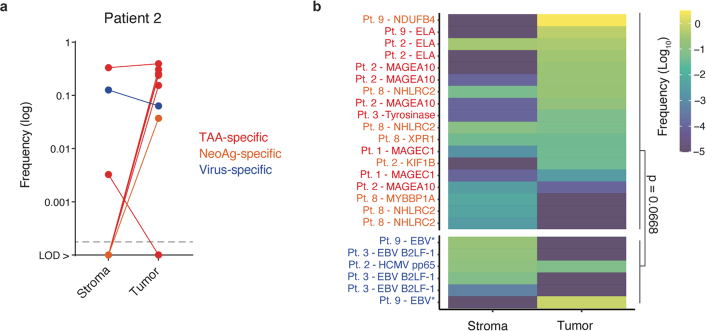
Microdissected stromal and tumor core areas. **a**) Representative example of the frequency of annotated CD8^+^ T-cell clones specific for TAAs, neoantigens or viral peptides in the microdissected stromal and tumor core areas of patient 2. Frequencies are displayed in logarithmic scale. **b**) Cumulative frequencies of annotated CD8^+^ T-cell clones specific for TAAs, neoantigens or viral peptides in the microdissected stromal and tumor core areas of five melanoma patients (1–5, Supplementary Table 3). Frequencies are displayed in logarithmic scale and statistics were performed using a one-tailed Chi-square test. Source data

We have previously reported that tumor-specific TIL clonotypes, especially those with high-avidity TCRs, accumulate preferentially within the tumor islets, that is, the tumor cell clusters circumscribed by surrounding stroma, while these clonotypes are largely diluted in the surrounding stroma^16,22^. To further challenge our prediction tools, we examined the spatial distribution of predicted TRTs. Taking advantage of TCR repertoires obtained from microdissected tumor core and stroma areas from five patients with melanoma with scRNA-seq/TCR-seq data (Fig. 2c and Supplementary Table 1), we first applied a predictor of TCR structural avidity^16^ and were able to validate the higher frequency of clones inferred to have high TCR avidity within the tumor islet compartment (Fig. 2d). Furthermore, by applying TRTpred, we found that TRTs were more frequent in tumor islets, while bystander TIL clones accumulated preferentially in the stroma (Fig. 2e and Extended Data Fig. 4a,b). This analysis was corroborated by analyzing previously identified^21^ neoantigen/tumor-associated antigen- or virus-specific T cells in a representative patient and in cumulative data (Extended Data Fig. 4a,b and Supplementary Table 3), validating the preferential infiltration of TRTs within tumor cores.

Besides exploratory applications, the ability to accurately distinguish TRTs (using TRTpred) represents an opportunity to identify private clinically relevant tumor-reactive TCRs for personalized TCR T cell therapy. For this purpose, two additional key qualitative features of TRTs must be considered to further select, among tumor-reactive clonotypes identified with TRTpred, the most clinically relevant clones. First, all TRTs may not be equally clinically relevant as they can be equipped with low-avidity TCRs, even if they target neoantigens^14,16^. Furthermore, in the perspective of personalized TCR-engineered T cell therapy using multiple TCRs, it is key to generate cell products targeting multiple distinct antigens to limit tumor escape^6,7^.

To this end, we propose MixTRTpred, a combinatorial algorithm that hinges on three steps: (1) applying TRTpred to generate a ranked list of tumor-reactive clones; (2) filtering out clones with inferred low structural avidity TCRs (Fig. 1a); and (3) applying a TCR clustering tool, that is, TCRpcDist^18^, to group TCRs with similar physicochemical properties into TCR-cluster, selecting the top tumor-reactive-scoring TCR in each cluster to maximize the diversity of targeted antigens. As a result, we obtain an optimized list of inferred tumor-reactive TCRs with high structural avidity targeting distinct antigens (MixTRTpred thus yields to an enrichment in clinically relevant TRTs but does not enhance, per se, the selection of tumor-reactive clonotypes).

**Extended Data Fig. 5:**
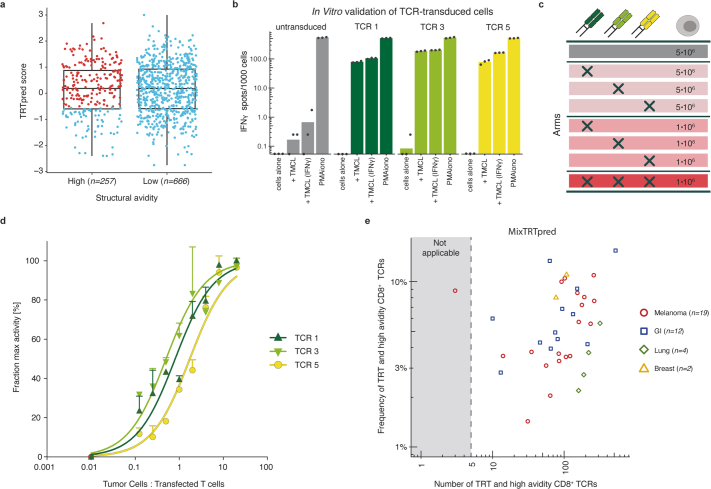
MixTRTpred clinical validation. **a**) TRTpred score distribution for predicted high and low avidity clones. Red points correspond to predicted tumor-reactive clones that are predicted as having high avidity. In box plots, the boxes represent the median and the interquartile range (IQR), while the whiskers extend to 1.5 times the IQR. **b**) *In vitro* validation of the selected three TCRs (TCR1, 3 and 5, Supplementary Table 6) for the *in vivo* experiment. T-cell responses of TCR-transfected cells against the autologous tumor cell line was assessed by IFNγ ELISpot assay (mean on n = 3 biologically independent replicates). **c**) Design of the *in vivo* experiment illustrating the different arms composed of products with individual or mixed TCR-transduced populations (TCR 1, 3 and 5, Supplementary Table 6). **d**) Dose response of Jurkat cells transfected with TCR 1, 3 or 5 and stimulated in different ratios with autologous tumor cells. Data show mean±SD on n = 2 biologically independent replicates. **e**) Total number and intratumoral frequency of clinically relevant clones (predicted to be tumor-reactive and of high avidity) in 37 patients from 4 datasets grouped by tumor-indications: Internal (melanoma, *n* = 14); Oliveira et al.^14^ (melanoma, *n* = 4); Hanada et al.^12^ (lung, *n* = 4); and Lowery et al.^8^ (*n* = 1 melanoma, *n* = 2 breast and *n* = 12 GI). A threshold of 5 distinct clinically relevant TCRs is shown. Colors correspond to the different tumor indications. Source data

To validate MixTRTpred’s efficacy, we applied it to a patient where autologous patient-derived xenograft tumors were available. From a total of 257 inferred high-avidity clones, we used TRTpred to select 188 TCRs predicted as tumor-reactive (Extended Data Fig. 5a). Then, from the 188 TRTs, we selected the top-ranking clonotypes (*n* = 20) and then used TCRpcDist^18^ to generate five distinct TCR clusters (Methods and Fig. 2f). By further selecting the top tumor-reactive TCRs from each cluster, we obtained five highly selected TCR candidates (Supplementary Table 6). Consistent with TRTpred’s overall accuracy, all TCRs (5 of 5) demonstrated tumor reactivity in vitro (Fig. 2g). We further selected three of these TCRs (spanning, agnostically, the whole range of in situ frequency) to investigate their potential in controlling the in vivo growth of autologous patient-derived xenograft tumors. Transfer of 5 × 10^6^ T cells engineered with each individual TCR controlled tumor growth (that is, 3 of 3; Fig. 2h and Extended Data Fig. 5b,c), and the two TCRs with the highest tumor reactivity scores and highest functional avidity eradicated tumors (Fig. 2h, Supplementary Table 6 and Extended Data Fig. 5d). To test the advantage of transferring multiple TCRs, we used a suboptimal T cell product (1 × 10^6^ cells per ACT dose) and compared the efficacy of cells transduced with only one of the individual TCRs (1–3) with a cocktail of all three TCRs. Multivalent ACT products yielded better tumor control than products containing only one type of TCR-transduced cells (Fig. 2i). Finally, applying MixTRTpred to all patients with assessable data (*n* = 37; Supplementary Table 1), we consistently identified more than ten clinically relevant clones per patient (median = 102), with the exception of one patient^14^ with only a few sequenced clones, thus demonstrating the applicability of MixTRTpred for TCR T cell therapy (Extended Data Fig. 5e).

In this study, we introduce TRTpred, a predictor of tumor-reactive clonotypes outcompeting existing tools in multiple datasets from different tumor indications. TRTpred enabled a granular interrogation of TIL repertoire and spatial distribution and revealed a large range of richness and clonality of TRT repertoires in tumors. These metrics, reflecting the abundance of tumor-reactive cells in tumors, may be useful in predicting responses to checkpoint immunotherapies, and behoove further studies to explore the utility of TRTpred in this field. Furthermore, preliminary observations indicate that the abundance of TRT in tumors predicts clinical responses to TIL ACT in melanoma^3^, offering further opportunities for better patient selection.

A minimum of ten distinct TCRs per patient was found using MixTRTpred. While more studies are needed to demonstrate the added value of the usage of multiple TCRs, the data reported here support the clinical relevance of inferred TCRs for adoptive immunotherapy.

Collectively, these observations suggest that TRTpred may be instrumental either to select patients who may benefit from TIL ACT or to select clinically relevant TCRs (using MixTRTpred) for TCR T cell therapy in remaining patients who would not be eligible for TIL therapy. Taking advantage of recent advances in the field of T cell engineering^6,23,24^, the accuracy of MixTRTpred indicates that personalized TCR-based therapy is now achievable for many patients with solid tumors.

## Methods

### Ethical statement

This research adheres to all applicable ethical regulations. Patient samples collected in this study were obtained following protocols approved by the institutional regulatory committee (Lausanne University Hospital, CHUV). Written, informed consent was obtained from all patients. In vivo experiments were performed in accordance with Swiss ethical guidelines and under approved licenses (see the next section), ensuring compliance with the 3R (replacement, reduction, refinement) guidelines.

### Cancer patient data collection

scRNA-seq/scTCR-seq data from tumors were obtained from internal patients with locally advanced (stage III) or metastatic (stage IV) cutaneous melanoma who had progressed on at least one standard first-line therapy. Tumor samples were obtained following surgery and processed as previously described^3,19^ for single-cell analysis. Briefly, scRNA-seq and scTCR-seq data were aligned to the GRCh38 reference genome using cellranger count (10X Genomics, v.3.0.1) and vdj (10X Genomics, v.3.1.0), respectively. We also applied TRTpred on external data from ref. ^14^ (melanoma, *n* = 4), ref. ^11^ (GI, *n* = 5), ref. ^8^ (*n* = 1 melanoma, *n* = 2 breast and *n* = 12 GI) and ref. ^12^ (lung, *n* = 4) collected from the Gene Expression Omnibus using SRA Toolkit (v.3.1.0). All data were processed and annotated following the authors’ guidelines. For the ref. ^8^ and ref. ^12^ datasets, neoantigen-specific clones were considered as tumor-reactive while the remaining clones were classified as non-tumor-reactive, adhering to the authors’ ‘closed-world’ assumption^8,12^. All patients are referenced in Supplementary Table 1.

### TCR cloning and tumor reactivity validation

TCRs from the ten internal melanoma patients were annotated following the rationale described in ref. ^3^. Briefly, TCR antitumor reactivity was interrogated by transferring RNA coding for TCRαβ pairs into recipient activated T cells and Jurkat cell line (TCR/CD3 Jurkat-luc cells (NFAT), Promega, stably transduced with human CD8α/β and TCRα/β CRISPR knockout). Electroporated cells were cocultured with interferon-γ (IFNγ)-treated autologous tumor cells and tumor reactivity was assessed through CD137 upregulation or bioluminescence assay for T cells and Jurkat cells, respectively. From the internal dataset (*n* = 10 patients), 102 tumor-reactive and 123 non-tumor-reactive CD8^+^ TCRs were used to build TRTpred and 46 tumor-reactive and 44 non-tumor-reactive CD8^+^ TCRs were used for the model benchmarking (*n* = 4 additional patients; Supplementary Table 1). Several TCRs were previously described^3^. For the dose–response curve, autologous activated T cells electroporated with TCR 1, 3 or 5 were cocultured with tumor cells in IFNγ assay, using precoated 96-well ELISpot plates (Mabtech) as described^16^.

### Statistical models to predict tumor reactivity

Two different approaches were used to predict cell-wise tumor specificity: the signature score and the LR approach. The models first predict cell-wise tumor specificity from scRNA-seq data which is then inferred on the TCR repertoire. The clone-wise score corresponds to the maximum tumor-reactive score obtained by any cell from a given clone.

The signature score approach, standard in RNA sequencing data analyses, uses differential expression analysis methods to derive a signature of tumor specificity which is then used to score cells. To allow comparison between the training and testing scores, the score is scaled based on the mean and standard deviation obtained from the training data. A threshold is then identified to stratify cells into tumor-specific and nonspecific cells, maximizing accuracy. Note that the score thresholds are identified in the training data and applied on the testing data. For this approach, nine different models were constructed upon the selection of nine differential expression analysis methods (DESeq2-Wald/-LRT, edgeR-QFL/-LRT, limma-trend/-voom, Seurat-LR/-negbinomial/-wilcox). These methods were carefully selected based on differential expression analysis benchmarking articles^25,26^ and applied following best practices (see the section ‘Differential expression analysis methods’). Other parameters such as how to select the genes from the differential expression analysis method (that is, by considering only the log-fold-change or the *P* value), the number of genes in the signature (that is, signature length), whether to take only the upregulated genes or both up- and downregulated genes (that is, signature side) and the signature score method (Average, AUCell, Singscore, UCell) were defined as hyperparameters to fine-tune (Extended Data Fig. 2a).

The LR approach uses a standard LR coupled with an elastic-net regularization. The type and strength of regularization are defined, respectively, by the *α* and *λ* hyperparameters, where *α* = 0 corresponds to Ridge regression and *α* = 1 to Lasso regression. From this definition, we derived LR models based on two different feature spaces: first the scaled expression data (RNA) and second the principal components. For the RNA feature space, the genes correlating more than 80% were removed. Because of the large dimensionality, and in addition to the elastic-net, we also tested filtering nonessential features. For both feature spaces, two different dimensionality reduction methodologies were used. The first consists of keeping only features associated with tumor specificity (Wilcoxon, *P* < 1%). For the RNA feature space, we also applied the differential expression analysis method mentioned above to keep only significant genes (Bonferroni-adjusted *P* < 0.05). Finally, another model was constructed solely on principal components, explaining more than 10^−4^ of the explained variances. The combination led to ten RNA- and two principal-components-based models (Extended Data Fig. 2a).

### Training and evaluation framework

The evaluations of the 21 models and their associated hyperparameter combinations were performed using an NCV (Extended Data Fig. 2b). This robust framework allows us to iteratively train and test the models on different data partitions called folds. For this application we used an LOPO NCV designed to partition the data into training folds composed of data from all patients but one, which constitutes the testing fold. Iteratively, this approach allows us to simulate the evaluation of the model on new unobserved data from other patients. For the sake of robustness, the performance metric chosen to evaluate the model is the reliable Matthews correlation coefficient (MCC)^27^. The LOPO evaluation serves as the final model evaluation, and the best model’s hyperparameters are fined tuned using a similar LOPO cross-validation. Ultimately, a final tumor specificity model is obtained by training the model using its best configuration on the whole input data.

### Signature collection and comparison

Five CD8^+^ TIL tumor-reactive signatures from different tumor indications were collected (refs. ^8,11,12,14,17^) and compared with TRTpred. All signatures have defined an upregulated gene-set but only refs. ^14^ and ^12^ have a downregulated gene-set. To compare two signatures, we have used Venn diagrams and the Jaccard index computed as the number of genes in common (intersection) divided by the total number of genes (union).

### Model benchmarking

To establish the robustness of the model’s association with tumor reactivity, we subjected the training and evaluation framework to y-randomization tests by applying the same methods with data composed of randomly permuted tumor-reactive annotated clones, repeating the process 100 times. This extensive analysis yielded an average MCC of 0 and an average accuracy of 50%, indicative of the model’s immunity to spurious learning and providing strong validation for its credibility. Encouragingly, we further validated the efficacy of our model on 100 and 205 CD8^+^ tumor-reactive annotated clonotypes, sourced from four internal and four external (ref. ^14^) melanoma tumor biopsies. To further explore the generalization potential of TRTpred, we also applied it on three external CD8^+^ TIL datasets from different tumor indications (ref. ^11^ (GI, *n* = 5), ref. ^8^ (melanoma, *n* = 1; breast, *n* = 2; GI, *n* = 12) and ref. ^12^ (lung, *n* = 4)). For the sake of completeness, we collected CD8^+^ TIL tumor-reactive signatures from these studies and applied them to each dataset. The external signatures were applied on each dataset by using the signature score method described in the respective studies. If not mentioned otherwise, a simple average signature score was computed. Finally, the discriminant power of the different signatures and TRTpred on each dataset was obtained by plotting receiver operating characteristic (ROC) curves and by computing the AUC for the ROC curves.

### Differential expression analysis methods

We applied nine different differential expression analysis methods found to work best in scRNA-seq data and applied them following best practices guidelines^26,28^. These methods were grouped into two classes: the methods developed for scRNA-seq data (LR, negative-binomial and Wilcoxon) and the methods originally developed for bulk RNA sequencing data (edgeR, DESeq2 and limma), named pseudo-bulk methods for the sake of clarity. The single-cell methods were implemented using the Seurat FindMarkers function on the log_10_-normalized unique molecular identifier counts with all filters (min.pct, only.pos, logfc.threshold, min.cells.group) disabled. To ensure the performance of the pseudo-bulk methods, we applied them on the clone-average log_10_-normalized unique molecular identifier counts. We did this to obtain a dataset resembling the bulk RNA sequencing data distribution (which reduces inconsistencies in pseudo-bulk methods^28^) while retaining the behavior of the clones transcription. EdgeR was applied both with the likelihood ratio test (edgeR-LRT, with default dispersion estimate) and with the quasi-likelihood approach (edgeR-QFL). DESeq2 was applied both with the Wald test of the negative-binomial model coefficients (DESeq2-Wald) and with a likelihood ratio test compared with a reduced model (DESeq2-LRT). Limma was applied using two approaches: one incorporating the mean-variance trend into the empirical Bayes procedure at the gene level (limma-trend) and the other incorporating the mean-variance trend by assigning a weight to each individual observation (limma-voom). The log-transformed counts per million values computed by edgeR were provided as input to limma-trend.

### Tumor microdissection and RNA extraction

Tumor microdissection and RNA extraction were performed as previously described^29^. Consecutive sections from fresh-frozen tissue blocks were cut in a cryostat at 8-μm thickness, mounted on precooled PET slides (Leica) at −20 °C for 1 h and fixed in ethanol. They were stained with cresyl violet, cleared in ethanol and microdissected within 20 min after staining using the Leica LMD7000. Laser parameters were set as follows: laser power of 39 mW, a wavelength of 349 nm, pulse frequency of 664 Hz, pulse energy of 58 μJ. Microdissected tissues were collected in 0.5-ml tubes in RNAlater solution (Thermo Fisher Scientific) and kept at −20 °C until RNA extraction. RNA was extracted using RNeasy Plus Micro Kit (Qiagen). To quantify total RNA, Qubit RNA HS Assay Kit and Qubit Fluorometer (Thermo Fisher Scientific) were used. The 2100 Bioanalyzer (Agilent) was used to analyze RNA fragment size using Eukaryote Total RNA Pico assay (Agilent).

### Sequential multiplexed immunohistochemistry

Sequential multiplexed immunohistochemistry was performed as previously described^29^. Fresh-frozen tissue sections were cut at 4 μm, fixed in paraformaldehyde (4%) overnight and permeabilized in 0.5% Triton X-100 in PBS for 30 min. After heat-mediated antigen retrieval in pH 6 citrate buffer for 10 min, endogenous peroxidases, nonspecific proteins, endogenous biotins and avidins were blocked (Dako). After application of the first primary antibody, a biotinylated secondary antibody and a streptavidin-HRP complex were added. Staining was revealed using AEC Chromogen. Tissues were counter-stained using Harris hematoxylin for 1 min and coated by a glass coverslip using an aqueous mounting solution. Slides were scanned into MRXS images using a Pannoramic 250 Flash III scanner (3D Histech). Glass coverslips were removed by immersion in hot water. AEC staining removed by immersion in ethanol of increasing concentrations. Antibodies were stripped by boiling tissue sections in a solution of citrate buffer (pH 6) for 10 to 20 min. Putative residual antibodies were blocked with Fab fragments. Multiplexed immunohistochemistry consisted of sequential cycles of: (1) staining with primary antibodies revealed by AEC Chromogen; (2) tissue section scanning; (3) removal of AEC Chromogen with ethanol; and (4) antibody stripping and blocking with Fab fragments. Primary antibodies were FOXP3 (clone ab99963, Abcam, dilution 1:50) and CD8 (clone C8/144B, Dako, dilution 1:20).

### Bulk TCR α and β sequencing

Bulk TCR sequencing analyses were performed as previously described^30^. Briefly, messenger RNA was isolated and amplified by in vitro transcription. A 5′ adapter was added by multiplex reverse transcription and TCRs were amplified using one primer in the adapter and one in the constant region. Libraries were sequenced on an Illumina instrument and TCR sequences processed using an ad hoc Perl script.

### TCR repertoire metrics

To analyze TCR repertoires, two metrics were used: (1) the richness corresponding to the total number of unique clones present in the repertoire and (2) the clonality described by the metric 1-Pielou’s evenness, as previously described^31^.

### MixTRTpred—integration of TCR structural avidity and TCR clustering

Predictions of TCR structural avidity were performed as described^32^. In brief, a binary LR, based on the CDR3β amino acids that are sufficiently solvent-exposed to interact with the cognate peptide, was used to determine whether a TCR was likely to bind the corresponding pMHC with high or low structural avidity (*k*_off_). Avidity levels were also computed with this model on assessable patients, that is, patients with scTCR-seq data composed of both alpha and beta CDR3 chain information. TCR clustering using TCRpcDist was performed as described^18^. TCRpcDist is a novel and fast structure-based approach that calculates similarities between TCRs using a metric related to the physicochemical properties of solvent-exposed amino acids of the most important residues of this receptor.

The TIL repertoire of patient 14 was analyzed and filtered through the three predictors, TRTpred, the high structural avidity predictor^32^ and TCRpcDist^18^. To combine the three axes, we first filtered out the low structural avidity predicted clones and ranked the resulting clones according to their tumor-reactive score. Finally, TCR clustering was applied on the subset of the top 20 tumor-reactive clones. The distance matrix obtained through TCRpcDist^18^ went through hierarchical clustering (agglomerative method: unweighted pair group method with arithmetic mean), leading to a dendrogram. We chose to split the latter into five distinct TCR clusters given the downstream in vitro and in vivo validation. The selection of five clusters was arbitrary and can be adapted depending on the clinical context.

### In vivo study

The in vivo study was performed as previously described^16^ and was approved by the Veterinary Authority of the Canton de Vaud (under license 3746) and performed in accordance with Swiss ethical guidelines. In brief, *Interleukin*-2 (IL-2) NOG mice (Taconic Biosciences) were monitored three times a week and given a score based on their weight, behavior, physical condition, dehydration, breathing and tumor burden. As per the protocol, animals reaching a defined score were killed.

### TCR transduction for in vivo experiment

TCRs 1, 3 and 5 were selected among the five in vitro-validated tumor-reactive TCRs from patient 14 (Supplementary Table 6) to be tested in vivo. The TCR transduction for the in vivo experiments was performed as previously described^16^. Briefly, primary CD8^+^ T cells from a healthy donor were negatively selected with beads (Miltenyi Biotec), activated and transduced as previously reported^16,33^. Transduced cells were stained with an APC-conjugated anti-mouse constant beta antibody (eBioscience), followed by sorting with anti-APC microbeads (Miltenyi Biotec). Sorted TCR-transduced CD8^+^ T cells were then expanded and tumor reactivity was assessed in IFNγ ELISpot assays (Mabtech). Transduced cells were then plated at 5 × 10^3^ cells per well and challenged with IFNγ-treated autologous tumor cells at a 1:1 ratio. After 18–20 h of incubation, cells were removed, the plate was developed according to the manufacturer’s instructions and cells were counted using a Bioreader 6000-E (BioSys).

### Adoptive T cell transfer in immunodeficient IL-2 NOG mice

The adoptive T cell transfer in immunodeficient IL-2 NOG mice was performed as previously described^16^. IL-2 NOG mice were subcutaneously injected with 10^6^ autologous human melanoma tumor cells from patient 14 and, once tumors became palpable (day 11), 1–5 × 10^6^ human tumor-specific CD8^+^ T cell clones were injected in the tail vein, according to the treatment arms described in Extended Data Fig. 5c, with 4–6 mice per condition except in some cases where 3 mice were considered.

### Data analyses and computation

Data analyses were performed using R Statistical Software (v.4.0.3). All data processing and analysis was performed using the R dplyr (v.1.1.0) and base libraries. The nested and simple cross-validations were performed using an in-house R library developed to control the models and hyperparameters throughout the folds. The R library glmnet (v.4.1-6) was used to build the LR models and their specifications. Parallelization of the computation was allowed using the foreach (v.1.5.2) library. The differential expression analysis methods were computed using the appropriate R libraries (Seurat (v.4.3.0), limma (v.3.50.3), edgeR (v.3.36.0), DESeq2 (v.1.34.0)) as well as the signature score methods (AUCell (v.1.16.0), UCell (v.1.3.1), singscore (v.1.14.0), GSEABase (v.1.56.0)). Statistical analyses were performed using the standard stats (v.4.1.2) library. The statistical tests used and their specifications are described in the figure legends. Parametric tests, for comparing two or more groups, were applied only on normally distributed variables validated with Anderson–Darling, D’Agostino–Pearson omnibus, Shapiro–Wilk and Kolmogorov–Smirnov tests (GraphPad v.9.1.0); otherwise, nonparametric tests were used.

### Plotting description

The figures were generated in R Statistical Software (v.4.0.3) with the ggplot2 (v.3.4.4) R package. Alluvial plots were generated using the ggalluvial (v.0.12.5) R package. The distance heatmaps were performed using the pheatmap function from the pheatmap (v.1.0.12) R package. Plotting of the scRNA-seq-derived UMAP was achieved using Seurat (v.4.3.0) R package functions. Venn diagrams were obtained using the ggven (v.0.1.10) R library. Schematic figures of T cells, cancer cells, TCRs, skin, lungs, intestine, breast, tumor and stromal microenvironment, plate and mouse in Fig. 1a were adapted from templates on BioRender.com. All figures were reprocessed using Adobe Illustrator 2023 (v.27.9.1) solely for esthetical purposes.

### Reporting summary

Further information on research design is available in the Nature Portfolio Reporting Summary linked to this article.

## Online content

Any methods, additional references, Nature Portfolio reporting summaries, source data, extended data, supplementary information, acknowledgements, peer review information; details of author contributions and competing interests; and statements of data and code availability are available at 10.1038/s41587-024-02232-0.

## Supplementary information

Supplementary Information Supplementary discussion and corresponding reference.

Reporting Summary

Supplementary Table 1 Cohorts’ description.

Supplementary Table 2 Model selection results.

Supplementary Table 3 Neoantigen, TAA and viral specificities.

Supplementary Table 4 TRTpred’ signature.

Supplementary Table 5 T-cell repertoire’s metrics for the 42 patients.

Supplementary Table 6 Description of the five TCR candidates assessed in vitro and in vivo.

## Source data

Source Data Fig. 1 Statistical source data.

Source Data Fig. 2 Statistical source data.

Source Data Extended Data Fig. 1 Statistical source data.

Source Data Extended Data Fig. 3 Statistical source data.

Source Data Extended Data Fig. 4 Statistical source data.

Source Data Extended Data Fig. 5 Statistical source data.

## Data Availability

scRNA-seq/scTCR-seq data from baseline tumors of patients 1–10/14 from the TIL ACT are available under the NCBI Gene Expression Omnibus (GEO) accession number GSE222448 (ref. ^34^). scRNA-seq/scTCR-seq data from baseline tumors of the additional melanoma patients 11, 12 and 13 are available at Zenodo via 10.5281/zenodo.10869332 (ref. ^35^). Source data are provided with this paper.

## Code availability

The computational code used in this study is proprietary and will be made available to academic researchers upon reasonable request. However, it has been assessed during the peer-review process.
