# Author Correction: Identification of clinically relevant T cell receptors for personalized T cell therapy using combinatorial algorithms

**DOI:** 10.1038/s41587-024-02318-9

**Published:** 2024-06-25

**Authors:** Rémy Pétremand, Johanna Chiffelle, Sara Bobisse, Marta A. S. Perez, Julien Schmidt, Marion Arnaud, David Barras, Maria Lozano-Rabella, Raphael Genolet, Christophe Sauvage, Damien Saugy, Alexandra Michel, Anne-Laure Huguenin-Bergenat, Charlotte Capt, Jonathan S. Moore, Claudio De Vito, S. Intidhar Labidi-Galy, Lana E. Kandalaft, Denarda Dangaj Laniti, Michal Bassani-Sternberg, Giacomo Oliveira, Catherine J. Wu, George Coukos, Vincent Zoete, Alexandre Harari

**Affiliations:** 1 Ludwig Institute for Cancer Research, Lausanne Branch, Department of Oncology, Lausanne University Hospital (CHUV) and University of Lausanne (UNIL), Agora Cancer Research Center, Lausanne, Switzerland; 2 Center for Cell Therapy, CHUV-Ludwig Institute, Lausanne, Switzerland; 3 Molecular Modelling Group, Swiss Institute of Bioinformatics (SIB), Lausanne, Switzerland; 4 Center of Experimental Therapeutics, Lausanne University Hospital (CHUV), Lausanne, Switzerland; 5 Department of Medicine and Center of Translational Research in Onco-Hematology, Faculty of Medicine, University of Geneva, Swiss Cancer Center Leman, Geneva, Switzerland; 6 Division of Clinical Pathology, Department of Diagnostics, Hôpitaux Universitaires de Genève, Geneva, Switzerland; 7 Department of Oncology, Hôpitaux Universitaires de Genève, Geneva, Switzerland; 8 Dana-Farber Cancer Institute, Boston, MA USA; 9 Harvard Medical School, Boston, MA USA; 10 Broad Institute of MIT and Harvard, Cambridge, MA USA; 11 Immuno-oncology Service, Department of Oncology, Lausanne University Hospital, Lausanne, Switzerland

**Keywords:** Tumour immunology, Applied immunology, Immunotherapy, Translational research, Computational models

Correction to: *Nature Biotechnology* 10.1038/s41587-024-02232-0, published online 7 May 2024

In the version of the article initially published, there were errors in Extended Data Fig. 5e. One patient was erroneously omitted due to an inaccurate *y*-axis scale, and in the key “*n* = 17” has been corrected to “*n* = 12”. These corrections have been made to the HTML and PDF versions of the article.

