# Supplementary material for: Identification of clinically relevant T cell receptors for personalized T cell therapy using combinatorial algorithms
Source: Nat Biotechnol. 2024 May 7;43(3):323–8. doi: 10.1038/s41587-024-02232-0 (PMC11919687; doi:10.1038/s41587-024-02232-0)
Supplement: Supplementary file 1 — Supplementary discussion and corresponding reference. [file 41587_2024_2232_MOESM1_ESM.pdf]

nature biotechnology

Brief Communication

https://doi.org/10.1038/s41587-024-02232-0

Identification of clinically relevant T cell receptors for personalized T cell therapy using combinatorial algorithms

In the format provided by the authors and unedited

Table of contents

Supplementary Discussion..*2*

References..*2*

Supplementary Discussion

TRTpred's accuracy is attributed to its training and evaluation framework, yet data quality and quantity are pivotal. For instance, in Oliveira *et al.*[1], TCRs were carefully selected from exhausted and naïve clusters which translated into highly effective TRT predictions. Furthermore, overfitting in the more complex LR model compared to the signature scoring were observed (**Supplementary Table 2**). Increasing TIL data quality and quantity may mitigate noise and enhance LR model performance. Moreover, enhancement of TRT prediction in non-melanoma tumors would require broadening the TILs input to be sourced in other tumors, for which data remain limited. Nonetheless, building single-cell predictive tools within more robust machine learning paradigm marks a vital step, not only in prediction of TRTs but also other possible phenotypes traits.

References

1. Oliveira, G. *et al.* Phenotype, specificity and avidity of antitumour CD8+ T cells in melanoma. *Nat. 2021* 1–7 (2021) doi:10.1038/s41586-021-03704-y.
