# Supplementary material for: Identification of clinically relevant T cell receptors for personalized T cell therapy using combinatorial algorithms
Source: Nat Biotechnol. 2024 May 7;43(3):323–8. doi: 10.1038/s41587-024-02232-0 (PMC11919687; doi:10.1038/s41587-024-02232-0)
Supplement: Supplementary file 2 — Reporting Summary [file 41587_2024_2232_MOESM2_ESM.pdf]

nature portfolio

Corresponding author(s): Alexandre Harari: alexandre.harari@chuv.ch

Last updated by author(s): Mar 28, 2024

Reporting Summary

Nature Portfolio wishes to improve the reproducibility of the work that we publish. This form provides structure for consistency and transparency in reporting. For further information on Nature Portfolio policies, see our Editorial Policies and the Editorial Policy Checklist.

Statistics

For all statistical analyses, confirm that the following items are present in the figure legend, table legend, main text, or Methods section.

n/a	Confirmed	
☐	☒	The exact sample size (*n*) for each experimental group/condition, given as a discrete number and unit of measurement
☐	☒	A statement on whether measurements were taken from distinct samples or whether the same sample was measured repeatedly
☐	☒	The statistical test(s) used AND whether they are one- or two-sided<br>*Only common tests should be described solely by name; describe more complex techniques in the Methods section.*
☐	☒	A description of all covariates tested
☐	☒	A description of any assumptions or corrections, such as tests of normality and adjustment for multiple comparisons
☐	☒	A full description of the statistical parameters including central tendency (e.g. means) or other basic estimates (e.g. regression coefficient) AND variation (e.g. standard deviation) or associated estimates of uncertainty (e.g. confidence intervals)
☐	☒	For null hypothesis testing, the test statistic (e.g. *F*, *t*, *r*) with confidence intervals, effect sizes, degrees of freedom and *P* value noted<br>*Give P values as exact values whenever suitable.*
☒	☐	For Bayesian analysis, information on the choice of priors and Markov chain Monte Carlo settings
☒	☐	For hierarchical and complex designs, identification of the appropriate level for tests and full reporting of outcomes
☒	☐	Estimates of effect sizes (e.g. Cohen's *d*, Pearson's *r*), indicating how they were calculated

Our web collection on statistics for biologists contains articles on many of the points above.

Software and code

Policy information about availability of computer code

Data collection

SRA Toolkit V.3.1.0 to collect data from the Gene Expression Omnibus protal (see data section bellow).

Data analysis

Data analyses and computation:
For internal data single-cell RNA-Seq analysis: Cell Ranger V.3.0.1, Seurat V3.
For internal data single-cell TCR-Seq analysis: Cell Ranger V.3.1.0
Data analyses were performed using R Statistical Software (V.4.0.3).
All data processing and analysis was performed using the R dplyr (V.1.1.0) and base libraries. The nested and simple cross-validation were performed using an in-house R library developed to control the models and hyper-parameters throughout the folds. The R library glmnet (V.4.1-6) was used to build the LR models and their specifications. Parallelization of the computation was allowed using the foreach (V.1.5.2) library. The differential expression analysis methods were computed using the appropriate R libraries (Seurat (V.4.3.0), limma (V.3.50.3), edgeR (V.3.36.0), DESeq2 (V.1.34.0)) as well as the signature score methods (AUCell (V.1.16.0), UCell (V.1.3.1), singscore (V.1.14.0), GSEABase (V.1.56.0)). Statistical analyses were performed using the standard stats (V.4.1.2) library. The statistical tests used, and their specifications are described in the figure legends. Parametric tests, for comparing two or more groups, were applied only on normally distributed variables validated with Anderson-Darling, D'Agostino-Pearson omnibus, Shapiro-Wilk and Kolmogorov-Smirnov tests (GraphPad V.9.1.0), otherwise, non-parametric tests were used.

Plotting description:
The figures were generated in R Statistical Software (V.4.0.3) with the ggplot2 (V.3.4.4) R package. Alluvial plots were generated using the ggalluvial (V.0.12.5) R package. The distance heatmaps were performed using the pheatmap function from the pheatmap (V.1.0.12) R package. Plotting of scRNA-seq derived UMAP was achieved using Seurat (V.4.3.0) R package functions. Venn diagrams were obtained using the ggven (V.0.1.10) R library. Schematic figures were created with BioRender.com. All figures were reprocessed using Adobe Illustrator 2023

(V.27.9.1) solely for esthetical purposes.

For manuscripts utilizing custom algorithms or software that are central to the research but not yet described in published literature, software must be made available to editors and reviewers. We strongly encourage code deposition in a community repository (e.g. GitHub). See the Nature Portfolio guidelines for submitting code & software for further information.

Data

Policy information about availability of data

All manuscripts must include a data availability statement. This statement should provide the following information, where applicable:

- Accession codes, unique identifiers, or web links for publicly available datasets
- A description of any restrictions on data availability
- For clinical datasets or third party data, please ensure that the statement adheres to our policy

ScRNA/TCR-Seq from baseline tumors of patients 1-10/14 is available under the NCBI Gene Expression Omnibus (GEO) accession number GSE222448. ScRNA/TCR-Seq from baseline tumors of the additional melanoma patients 11, 12 and 13 is available under the zenodo DOI: 10.5281/zenodo.10869332.

We collected single-cell RNA/TCR sequencing FastQ files from 3 external cohort: (i) Zheng et al., 2022, Cancer Cell 40, 410–423, (ii) Hanada et al., 2022, Cancer Cell 40, 479–493 & (iii) Lowery et al., 2022, Science 375, 877–884. This data was collected through data-mendeley (for i) and through the database of Genotypes and Phenotypes (dbGaP) using SRA toolkit.

Research involving human participants, their data, or biological material

Policy information about studies with human participants or human data. See also policy information about sex, gender (identity/presentation), and sexual orientation and race, ethnicity and racism.

Reporting on sex and gender	No bias induced by gender or age was present in patients enrolment. Information about the age and gender can be found in Supplementary Table 1.
Reporting on race, ethnicity, or other socially relevant groupings	Race, ethnicity or other socially relevant groupings were not considered.
Population characteristics	All internal patients were adults with histologically proven unresectable locally advanced or metastatic melanoma. All patients had progressed on at least one standard first line therapy, including but not limited to chemotherapy, BRAF and MEK inhibitors, anti-CTLA4, anti-PD1 or anti-LAG3 antibodies and/or the combination. Information about the age and gender can be found in Supplementary Table 1.
Recruitment	Patients were enrolled under protocols approved by the respective institutional regulatory committees at the Lausanne University Hospital (Switzerland).
Ethics oversight	Recruitments and procedures were approved by regulatory authorities of the Lausanne University Hospital (Switzerland) and all patients signed written informed consents.

Note that full information on the approval of the study protocol must also be provided in the manuscript.

Field-specific reporting

Please select the one below that is the best fit for your research. If you are not sure, read the appropriate sections before making your selection.

☒ Life sciences ☐ Behavioural & social sciences ☐ Ecological, evolutionary & environmental sciences

For a reference copy of the document with all sections, see nature.com/documents/nr-reporting-summary-flat.pdf

Life sciences study design

All studies must disclose on these points even when the disclosure is negative.

Sample size	All in vitro studies were performed according to sample availability (when using transfected PBMCs). When not available or for results confirmation, a Jurkat cell line (TCR/CD3 Jurkat-luc cells (NFAT), Promega, stably transduced with human CD8α/β and TCRα/β CRISPR-KO) was used. No sample-size calculation was performed.<br>For in vivo studies, 1 to 5 millions CD8 T cells/mouse were used. We achieved a sample size of 3 to 5 animals per treatment group to be able to reproducibly observe statistically significant differences. Sample size was not predetermined and sample size of 3 to 5 animals per conditions was chosen to illustate the relative efficacy of the different TCRs or combination of the different TCR. No sample-size calculation was performed.
Data exclusions	No data were excluded in this study.
Replication	To validate TCR reactivity in vitro, n=2 biologically independent replicates was used for unambiguously clear responses. Both negative and positive controls were consistently included. To validate the TCR-transfected T-cell responses against the autologous tumor cell line, n=3

biologically independent replicates were used. All attempts were successful. Both negative and positive controls were consistently included. The sequential multiplexed immunohistochemistry, tumor microdissection and RNA extraction were performed only once.

Randomization	For most analyses all patients were considered. For figures 2c-e and extended data Figure 4a-b the patients were randomly selected based on sample availability. No other covariate was considered.
Blinding	To minimize stress to the mice, treatment administration and tumor volume measurements were made at the same time (during same anaesthesia). An independent investigator verified caliper measurements in a blinded fashion. Analysis of data (plotting of pre-recorded tumor volumes at end of study) was performed in a non-blinded manner.

Reporting for specific materials, systems and methods

We require information from authors about some types of materials, experimental systems and methods used in many studies. Here, indicate whether each material, system or method listed is relevant to your study. If you are not sure if a list item applies to your research, read the appropriate section before selecting a response.

Materials & experimental systems

n/a	Involved in the study	
☒	☐	Antibodies
☐	☒	Eukaryotic cell lines
☒	☐	Palaeontology and archaeology
☐	☒	Animals and other organisms
☒	☐	Clinical data
☒	☐	Dual use research of concern
☒	☐	Plants

Methods

n/a	Involved in the study	
☒	☐	ChIP-seq
☒	☐	Flow cytometry
☒	☐	MRI-based neuroimaging

Eukaryotic cell lines

Policy information about cell lines and Sex and Gender in Research

Cell line source(s)	Tumor lines were derived from tumor biopsies in-house. TCR/CD3 Jurkat-luc cells (NFAT) stably transduced with human CD8α/β and TCRα/β CRISPR-KO were obtained from Promega. 293T cells were obtained from ATCC.
Authentication	Internal tumor cell lines and TCR/CD3 Jurkat were not authenticated.
Mycoplasma contamination	Before each study, cells were tested negative for mycoplasma by PCR in-house.
Commonly misidentified lines (See ICLAC register)	No misidentified cell lines were used.

Animals and other research organisms

Policy information about studies involving animals; ARRIVE guidelines recommended for reporting animal research, and Sex and Gender in Research

Laboratory animals	Interleukin-2 (IL-2) NOG mice, constitutively expressing human IL-2, were obtained from Taconic Biosciences and maintained in a conventional animal facility at the University of Lausanne under specific pathogen-free status, with dark/light cycles of 12 h, humidity 55% (±10%) and a temperature of 22 °C (±1 °C). Twenty to twenty-five-week-old male and female mice (randomly selected) were used in the experiment.
Wild animals	No wild animals were used in this study.
Reporting on sex	Sex of animals was not considered in this study.
Field-collected samples	No field-collected samples were used in this study.
Ethics oversight	This study was approved by the Veterinary Authority of the Canton de Vaud (under the license VD3746) and performed in accordance with Swiss ethical guidelines.

Note that full information on the approval of the study protocol must also be provided in the manuscript.

Plants

Seed stocks	*Report on the source of all seed stocks or other plant material used. If applicable, state the seed stock centre and catalogue number. If plant specimens were collected from the field, describe the collection location, date and sampling procedures.*
Novel plant genotypes	*Describe the methods by which all novel plant genotypes were produced. This includes those generated by transgenic approaches, gene editing, chemical/radiation-based mutagenesis and hybridization. For transgenic lines, describe the transformation method, the number of independent lines analyzed and the generation upon which experiments were performed. For gene-edited lines, describe the editor used, the endogenous sequence targeted for editing, the targeting guide RNA sequence (if applicable) and how the editor was applied.*
Authentication	*Describe any authentication procedures for each seed stock used or novel genotype generated. Describe any experiments used to assess the effect of a mutation and, where applicable, how potential secondary effects (e.g. second site T-DNA insertions, mosiacism, off-target gene editing) were examined.*
